# The African American United Memory and Aging Project: AD Knowledge and Family History as It Relates to Cognition

**DOI:** 10.1093/geroni/igab046.371

**Published:** 2021-12-17

**Authors:** Alyssa Gamaldo, Allison Caban-Holt, Travonia Brown-Hughes

**Affiliations:** 1 The Pennsylvania State University, University Park, Pennsylvania, United States; 2 Maya Angelou Center for Health Equity, Winston-Salem, North Carolina, United States; 3 School of Pharmacy, Hampton, Virginia, United States

## Abstract

This study explores the influence of Black adults’ Alzheimer’s disease (AD) knowledge and family history of AD on cognition. A sample of Black adults (n=66, age range=45-84) completed a computerized cognitive (CogState Brief) battery and surveys of AD knowledge, family history of AD diagnosis, and health. On the 14-item AD knowledge survey, participants correctly answered a mean of 10.80 (SD=1.50) items. Approximately, 56% reported a biological family member diagnosed with AD, of these 30% reported this being a mother or father. Linear regression models suggested no significant association between AD knowledge and cognitive performance. However, adults with a family member diagnosed with AD had worse visual learning accuracy even after adjusting for age, education, and income. Increased age was associated with worse processing speed, particularly in adults with a mother diagnosed with AD. These findings demonstrate the importance of examining the influence of family history on Black adults’ cognitive health.

